# Eotaxin and FGF enhance signaling through an Extracellular signal-related kinase (ERK)-dependent pathway in the pathogenesis of Eosinophilic Esophagitis

**DOI:** 10.1186/1710-1492-6-25

**Published:** 2010-09-05

**Authors:** Jennifer J Huang, Jae Won Joh, Judy Fuentebella, Anup Patel, Tammie Nguyen, Scott Seki, Lisa Hoyte, Neha Reshamwala, Christine Nguyen, Anthony Quiros, Dorsey Bass, Eric Sibley, William Berquist, Kenneth Cox, John Kerner, Kari C Nadeau

**Affiliations:** 1 Stanford School of Medicine, Stanford, CA 94305, USA; 2 California Pacific Medical Center, San Francisco, CA 94118, USA

## Abstract

**Background:**

Eosinophilic esophagitis (EoE) is characterized by the inflammation of the esophagus and the infiltration of eosinophils into the esophagus, leading to symptoms such as dysphagia and stricture formation. Systemic immune indicators like eotaxin and fibroblast growth factor were evaluated for possible synergistic pathological effects. Moreover, blood cells, local tissue, and plasma from EoE and control subjects were studied to determine if the localized disease was associated with a systemic effect that correlated with presence of EoE disease.

**Method:**

Real-time polymerase chain reaction from peripheral blood mononuclear cells (PBMC), immunohistochemistry from local esophageal biopsies, fluid assays on plasma, and fluorescence-activated cell sorting on peripheral blood cells from subjects were used to study the systemic immune indicators in newly diagnosed EoE (n = 35), treated EoE (n = 9), Gastroesophageal reflux disease (GERD) (n = 8), ulcerative colitis (n = 5), Crohn's disease (n = 5), and healthy controls (n = 8).

**Result:**

Of the transcripts tested for possible immune indicators, we found extracellular signal-regulated kinase (ERK), Bcl-2, bFGF (basic fibroblast growth factor), and eotaxin levels were highly upregulated in PBMC and associated with disease presence of EoE. Increased FGF detected by immunohistochemistry in esophageal tissues and in PBMC was correlated with low levels of pro-apoptotic factors (Fas, Caspase 8) in PBMC from EoE subjects. Plasma-derived bFGF was shown to be the most elevated and most specific in EoE subjects in comparison to healthy controls and disease control subjects.

**Conclusion:**

We describe for the first time a possible mechanism by which increased FGF is associated with inhibiting apoptosis in local esophageal tissues of EoE subjects as compared to controls. Eotaxin and FGF signaling pathways share activation through the ERK pathway; together, they could act to increase eosinophil activation and prolong the half-life of eosinophils in local tissues of the esophagus in EoE subjects.

## Introduction

Eosinophilic esophagitis (EoE) is an inflammatory disorder of the esophagus that can be characterized by feeding difficulties, heartburn, regurgitation, vomiting, abdominal pain, dysphagia, and food impaction [1-3]. EoE seems to predominantly affect children, and in particular, males [4]. Epidemiologic data from 2008 suggest that in children the annual incidence of EoE is approximately 1.3-1.6 per 10,000 and the prevalence is approximately 4.3-9.1 per 10,000 [5,6]. Approximately 50% of subjects with EoE have co-existing atopic conditions, such as food allergy, asthma, or eczema [7-12]. Food allergy is particularly prominent, but aeroallergens also may play a role [13].

The clinical symptoms of EoE are often similar to those of gastroesophageal reflux disease (GERD) and initial illness may be thought to be GERD. However, the symptoms do not resolve with gastric acid suppression, and EoE presents with esophageal abnormalities during endoscopy, showing eosinophil infiltration in the esophagus. EoE is currently defined as the presence of more than 15 intraepithelial eosinophils per high-powered field (eos/HPF) in both the proximal and distal esophagus [14]. Endoscopy of the subjects often shows signs of longitudinal linear furrows, trachealization, white plaques, and strictures but usually with a negative pH probe result. Subjects with more severe disease status present with severe stricturing, furrowing, and/or trachealization, which may lead to mechanical dilation of the esophagus or food impaction that requires surgical removal [13].

EoE diagnosis can only be made through endoscopy and biopsy of esophageal tissue. Prognostic tests that are easily obtained and efficiently run through minimally invasive techniques to predict disease progression are lacking. Current guidelines suggest continual biopsies for monitoring of disease progress and treatment efficiency [15]. Since endoscopy with biopsy entails risks to patients, we aimed to study immune indicators found in plasma as secreted proteins that correlate with local presence in esophageal tissues in EoE subjects as compared to controls. Moreover, we hypothesized that through the discovery of specific EoE immune indicators, we could further interrogate pathological mechanisms of EoE disease. Specifically, we focused on examining immune indicators associated with EoE, for example, eotaxin and FGF. FGF is a protein involved in cell development, cell differentiation and tissue repair [16]. Additionally, bFGF has been shown to enhance the half-life of cells [17] and both bFGF and eotaxin coordinate their activity through the activation of ERK [18,19]. Thus, in particular, we hypothesized that FGF could enhance activation and half-life of eosinophils in local esophageal tissues as compared to peripheral blood.

Altogether, the data presented here provide a mechanistic explanation for how basic fibroblast growth factor could worsen the pathogenesis of EoE by enhancing activation of eosinophils through synergy with eotaxin via the ERK signaling pathway. The data may be used in the possible diagnosis and prognosis of EoE. In addition, our results could provide an explanation for increased eosinophil numbers, and prolongation of eosinophil half-life in EoE disease pathology.

## Methods

### Human Clinical Data collection

The study was approved by the Stanford Administrative Panel on Human Subjects in Medical Research. All subjects signed informed consent forms before participating in the study. The study was performed according to Declaration of Helsinki guidelines. Proximal and distal esophageal tissue and peripheral blood were obtained from subjects during a diagnostic endoscopy and subsequent biopsy. Middle esophageal biopsy tissue was also obtained from some patients. Biopsy samples were preserved as formalin-fixed and paraffin-embedded sections. Histological diagnosis of EoE, GERD, or other disorders (i.e, ulcerative colitis and Crohn's disease) was made by a licensed and certified clinical pathologist in gastroenterology at Stanford University School of Medicine Clinical laboratory using guidelines by calculating the mean number of eosinophils per high power field (HPF) in more than 3 areas per biopsy sample [7]. Subjects who had no pathological basis for symptoms and who had a negative pH probe were considered healthy controls (HCs). Subjects who had a negative pH probe result and whose histological sections contained greater than 15 eos/HPF were diagnosed with EoE as per guidelines [14,20]. All EoE subjects had failed a trial of proton pump inhibitor therapy (specifically, for an average of 6-8 weeks of therapy) before being diagnosed with EoE. Subjects with a positive pH probe test and a negative biopsy result were diagnosed with GERD. GERD subjects had less than 6 eosinophils/HPF in the esophagus on biopsy. Subjects labeled as "treated EoE" were confirmed with biopsies of esophagus showing improvement (less than 5-10 eosinophils/HPF) and demonstrated resolution of clinical symptoms. Subjects with eosinophilic gastritis, eosinophilic colitis, acute or chronic infections (viral, bacterial or fungal), autoimmune disease, or neoplasm were excluded from the EoE, HC, and GERD groups. Allergic subjects were defined as having a total serum IgE of >25 IU/ml and positive skin prick testing as compared with positive histamine control to foods. Patients were not allergy patch-tested routinely. None of the subjects had active infections and no subjects had positive findings for parasitic infections. Overall, samples were obtained on newly diagnosed EoE (n = 35), treated EoE (n = 9), GERD (n = 8), ulcerative colitis (UC, n = 5), and Crohn's disease (CD, n = 5), healthy controls (n = 8) respectively. Demographics for each subject are found in Additional File 1, Table S1.

### Plasma and PBMC separation

Each blood sample was centrifuged at 1800 RPM for 10 minutes, and the supernatant plasma layer was collected and centrifuged for another 10 minutes at 13,000 RPM. Plasma was stored in 250 μl aliquots at -80°C. Phosphate-buffered saline (PBS) was added to the remaining blood cell layer until the total volume was double the original collected blood volume. The homogeneous PBS-blood mixture was layered over the Ficoll reagent (MP Biomedicals, Solon, OH) at a 2:1 volume ratio and subsequently centrifuged at 2200 RPM for 20 minutes. The peripheral blood mononuclear cell (PBMC) layer was removed and washed twice with PBS at 1800 RPM for 5 minutes. PBMCs were then re-suspended at 1 million cells per milliliter in a solution of 90% FBS and 10% DMSO and stored for later use for QT-PCR.

### Fluid Assays

Plasma samples underwent testing with Luminex 35-plex technology (Invitrogen, Carlsbad, CA). 35 cytokines and chemokines were assayed: fibroblast growth factor basic (bFGF or FGF-2); eotaxin (1, 2, and 3); IL-1α; IL-1β; IL-1 receptor antagonist (IL-1RA); IL-2; IL-4; IL-5; IL-6; IL-7; IL-8; IL-10; IL-12-p40; IL-12-p70; IL-13; IL-15; IL-17; IL-17F; epithelial cell-derived neutrophil-activating protein-78 (ENA78); granulocyte colony-stimulating factor (G-CSF); granulocyte-macrophage colony-stimulating factor (GM-CSF); growth-related oncogene-alpha (GRO-α); interferon-gamma (IFN-γ); interferon-inducible protein 10 (IP10); leptin; monocyte chemotactic protein-3 (MCP-3); monokine induced by gamma interferon (MIG); macrophage inflammatory protein 1 alpha (MIP-1α); macrophage inflammatory protein 1 beta (MIP-1β); nerve growth factor (NGF); platelet-derived growth factor-BB (PDGF-BB); regulated upon activation, normal T cell expressed and secreted (RANTES); transforming growth factor beta (TGF-β); tumor necrosis factor alpha (TNF-α); tumor necrosis factor beta (TNF-β), and vascular endothelial growth factor (VEGF). Samples were tested and normalized with standard curves to ensure consistency and calibrations occurred before each run, per manufacturer's instructions (Luminex Technologies, Invitrogen, Carlsbad, CA). Furthermore, each sample was run in duplicate for quality control. In addition, some plasma samples (n = 10) from subjects were run through Cytometric Bead Array (CBA) Multiplex technology (BD Biosciences, San Jose, CA; Th1/Th2 cytokine Assay, used per manufacturer's instructions) to test the reproducibility of the Luminex 35-plex technology. For those cytokines tested (i.e., eotaxin-3, basic fibroblast growth factor (bFGF), G-CSF, IFN-", IL-17, IL-1α, IL4, IL-5, macrophage inflammatory protein 1α, and nerve growth factor), similar results were obtained with CBA and Luminex technologies.

### Immunohistochemistry

Paraffin-embedded tissue samples were soaked in xylene and then solutions of 100%, 95%, and 70% ethanol sequentially to remove the paraffin wax. Antigen unmasking was performed by heating the slides in a decloaking chamber to 120°C in Diva Decloaking buffer (Biocare Medical, Concord, CA), and then cooling to room temperature. H_2_O_2 _block (Lab Vision, Fremont, CA) and protein block (Dako, Glostrup, Denmark) were then applied to the tissue to prevent non-specific binding and block endogenous peroxidases. Unconjugated mouse anti-human FGF-9 primary antibody (Clone D-8) (Santa Cruz Biotechnology) was diluted 1:50 and applied for 2 hours at 25°C. After washing, a secondary MACH 2 Mouse HRP (Biocare Medical, Concord, CA) antibody was applied for 30 minutes. After further washing, the slides were stained with DAB (Vector Labs, Burlingame, CA) and subsequently counterstained with hematoxylin before being mounted. Cells were counted per high-powered field (HPF, 400×) at three different sites in the tissue and the mean HPF was calculated. Polyclonal unconjugated rabbit anti-human FGF-2 primary antibody (Abcam, Cambridge, MA) was diluted 1:250 with antibody diluent (Dako, Glostrup, Denmark) and was double-stained with mouse anti-human EG2 primary antibody (gift of Dr. Reinhard B. Raggam). A MACH 2 Double Stain HRP-AP secondary antibody (Biocare Medical, Concord, CA) was used.

### QT-PCR RNA analysis

PBMCs from each subject were used for QT-PCR RNA analysis. For cDNA synthesis, 500 ng total RNA was transcribed with cDNA transcription reagents (Applied Biosystems, Foster City, CA) using random hexamers, according to the manufacturer's instructions. Gene expression was measured in real-time with the GeneAmp 7900 Sequence Detection System (Applied Biosystems, Foster City, CA) using primers and other reagents purchased from Applied Biosystems. Relative quantification was measured using the Comparative CT (Threshold Cycle) method. The expression level of a gene in a given sample was represented as 2^-ΔΔ Ct ^where ΔΔCT = [ΔCT_(experimental)_] - [ΔCT_(medium)_] and ΔCT = [CT_(experimental)_] - [CT_(housekeeping)_]. All PCR assays were performed in triplicate. 100 ng of total isolated RNA was submitted to reverse transcription (Invitrogen, Carlsbad, CA). 50 ng of resulting cDNA was submitted to TaqMan™PCR on an ABI Systems qPCR machine (Applied Biosystems, Foster City, CA) at the Stanford Department of Pediatrics QT-PCR Facility using gene-specific, fluorochrome-labeled probe/primer sets purchased from Applied Biosystems, Inc. EoE, GERD, HC, UC, and CD samples were tested for fibroblast growth factor (FGF), fibroblast growth factor receptor (FGF-R), eotaxin, C-C chemokine receptor type 3 (CCR3), extracellular signal-regulated kinase (ERK), c-Jun N terminal kinase (JNK), IL-13, IL-5, Fas, B cell lymphoma protein 2 (Bcl-2), and normalized to the β-2-microglobulin gene.

### Statistics

Statistical analysis was performed with the GraphPad Prism software (GraphPad Software, La Jolla, CA). Statistical comparisons of data among groups were performed using the one-way analysis of variance (ANOVA) non-parametric Kruskal-Wallis test and the Dunn's Multiple Comparison post-test. Differences were considered significant at a p-value of less than or equal to 0.05. Correlation analysis between fold expressions was performed using the Spearman correlation.

## Results

### Demographics

Data were analyzed from newly diagnosed EoE (n = 35), treated EoE (n = 9), GERD (n = 8), ulcerative colitis (UC, n = 5), and Crohn's disease (CD, n = 5), healthy controls (HC, n = 8) respectively. Demographics for each subject are found in Additional File 1, Table S

1. Consistent with past findings, EoE seemed to particularly affect males (25/35 subjects). Subjects (71%) had elevated IgE levels and positive prick skin testing to at least one food allergen (performed at the Stanford Allergy and Immunology Clinics). None of the UC or CD subjects were on medications at the time of biopsy since these were representative newly diagnosed subject biopsies for the UC and CD subjects.

Prior to the subjects' endoscopy for possible diagnosis of EoE, subjects had been treated with anti-reflux therapy for 6-8 weeks without a positive response. Seven subjects were treated with only swallowed budesonide, 6 were treated with only swallowed fluticasone, 3 were treated with only an elimination diet, 6 were treated with only an elemental diet, and 11 were treated with both swallowed budesonide and an elimination diet (Additional File 1, Table S2).

### QT-PCR Assays demonstrate differential expression of immune indicators in EoE

**Figure 1:**
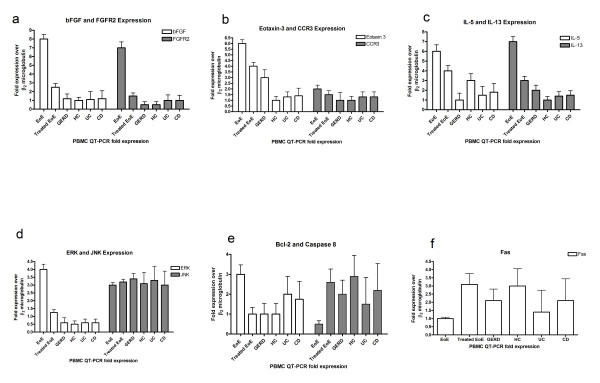
**a-f The fold expression of immune indicators bFGF, FGF-R, Eotaxin-3, CCR3, IL-5, IL-13, ERK, JNK, Bcl-2, Capase 8, and Fas**. a) EoE subjects statistically significant increases in levels of bFG and FGFR2 (p < 0.001) There was an 8-fold increase in the bFGF levels in EoE subjects as compared to HC subjects and a 4-fold increase compared to GERD; b)There was a 6-fold increase in eotaxin-3 comparing EoE subjects to HC and a 2-fold increased when compared to GERD subjects. However, this was not statistically significant. CCR3 levels were consistent among all subject groups; c) There was a statistically significant increase in the amount of IL-5 in subjects with EoE as compared to GERD (p < 0.01). Increase in IL-13 in EoE was statistically significant compared to all other subject groups (p < 0.001); d) ERK was high increased compared to other subjects groups (p < 0.001) while JNK levels remained consistent; e) EoE subjects had a 3-fold increase of Bcl-2 and a 3-fold decrease in caspase 8 expression compared to treated EoE subjects (p < 0.05); f) Fas expression was down-regulated by 3-fold in EoE subjects as compared to treated EoE subjects (p < 0.05). EoE: Eosinophilic Esophagitis; GERD: gastroesophageal reflux disease; HC: healthy control; UC: ulcerative colitis; CD: Crohn's disease.

RNA purified from subject PBMCs was used for QT-PCR assays. Transcripts for bFGF were found to be increased in newly diagnosed EoE subjects and expression was decreased after treatment. QT-PCR data showed that untreated EoE subjects had an increased expression of bFGF that was up to eight-fold higher (n = 35) compared to the HCs (n = 8, 1×, p < 0.05), and compared to when on treatment (n = 9 EoE subjects, 1.5×, p < 0.001). Although FGF levels in GERD subjects were slightly elevated (n = 8), it was not statistically significant compared to HC (p > 0.05) (Figure 1a). EoE subjects had statistically significant increased FGFR2 expression compared to HC (14×, p < 0.05) (Figure 1a). EoE subjects had a slight but not statistically significant increase in eotaxin-3 expression when compared to GERD (2×, p > 0.05) and to HC (6×, p > 0.05) (Figure 1b). Eotaxin-1 and 2 transcripts were also assessed and found similar to eotaxin-3 expression patterns (data not shown). CCR3, the eotaxin-3 receptor, also had an increased expression factor of 2× compared to GERD and HC (Figure 1b). IL-5 expression was a six-fold increase in EoE subjects versus GERD and a three-fold increase versus HC (Figure 1c). However, the difference was not statistically significant. IL-13 had a statistically significant seven-fold increase in EoE subjects versus HC (p < 0.05) and a 3.5 fold increase over GERD subjects (p < 0.001) (Figure 1c). IL-13 increases were associated with EoE subjects with concomitant food allergies (data not shown).

**Figure 2:**
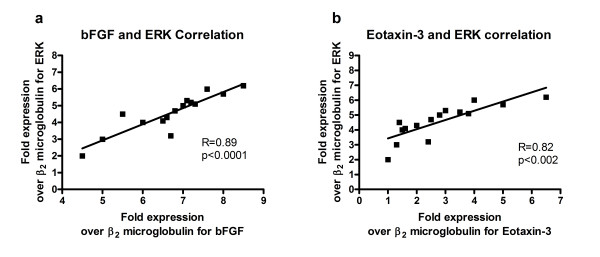
**a There is a positive correlation between the fold expression of bFGF and ERK, suggesting that the upregulation of bFGF may also influence the upregulation of ERK**. Figure 2b: There is a positive correlation between the fold expression of ERK and eotaxin-3.

In summary, specific immune indicators with increased expression in newly diagnosed EoE compared to treated EoE, HC, GERD, UC, and CD were: bFGF, FGF-Receptor 2, IL-13, IL-5, eotaxin, and CCR3 (in order of highest to lowest extent of increased transcript expression). Interestingly, upon examination of transcript expression of signaling pathway proteins and apoptosis-related proteins such as ERK, JNK, Bcl-2, caspase 8, and Fas, for the newly diagnosed EoE subjects, it was found that the signaling pathway transcripts of ERK but not JNK were found to be increased relative to HC, UC, CD and treated EoE (Figure 1d). ERK was expressed at a minimum of four-fold increase at statistically significant differences compared to all other subjects (p < 0.05) (Figure 1d). The extent of the increases in ERK was correlated with bFGF increases in expression (R = 0.89, p < 0.05) (Figure 2a) and with eotaxin increases in expression (R= 0.82, p < 0.05) (Figure 2b). This suggests that FGF signaling could occur through ERK but not JNK, and that this signaling could enhance the ERK-dependent signaling pathways associated with eotaxin. EoE subjects had a 3-fold increase in Bcl-2 expression compared to treated EoE patients while caspase 8 showed a 3-fold decrease (p < 0.05) (Figure 1e). Likewise, there was a 3-fold decrease in Fas expression levels in EoE subjects compared to the treated EoE subjects (p < 0.05) (Figure 1f).

### Fluid Assays on plasma show highly elevated bFGF specifically in EoE subjects

**Figure 3:**
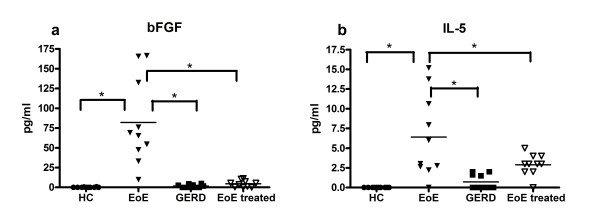
a) EoE subjects had an elevated level of bFGF in comparison (p < 0.05) to those of HC, GERD, and treated EoE subjects (same subjects as EoE subject but now on therapy); b)IL-5 was also elevated in EoE subjects compared to treated EoE subjects (p < 0.05) and gastroesophageal reflux disease and healthy control subjects (p < 0.05). (*p < 0.05).

Since we found bFGF and other immune indicators to be specifically increased in PBMCs of EoE subjects, we then performed fluid assays to determine whether they were present systemically in the plasma. Our fluid assay data (performed via Luminex 35-plex technology per manufacturer's instructions, Invitrogen and via Cytometric Bead Array Technology per manufacturer's instructions, BD Biosciences) were important in the narrowing of potential immune indicators specific to eosinophilic esophagitis. In plasma from HC subjects (n = 10), bFGF levels were low (mean 0.13 pg/mL, s.e. 0.09 pg/mL) while bFGF was significantly upregulated in EoE subjects (n = 10, mean 81.98 pg/mL, s.e. 17.23 pg/mL for EoE, p < 0.05). Differences in bFGF levels between EoE and GERD were also significant (n = 10, mean 1.74 pg/mL, s.e. 0.64 for GERD, p < 0.05) (Figure 3a). Treated EoE subjects had a statistically significant decrease in bFGF (n = 9, mean 4.60 pg/mL, s.e. 1.35 pg/mL, p < 0.05) (Figure 3a). Treated EoE subjects were the same subjects as the EoE subjects. A decrease in bFGF was seen in all subjects. There was a significant difference in the level of peripheral IL-5 in EoE subjects compared to HC (p < 0.05), GERD (p < 0.05), and EoE treated (p < 0.05) (Figure 3b). Other cytokines that showed statistically significant differences between HC and EoE subjects include G-CSF, GRO-α. IFN-γ, IL-15, IL-13, IL-17, IL-2, IP-10. MIG, MIP-1α, MIP-1β, NGF, and RANTES (data not shown).

### Immunohistochemistry

**Figure 4:**
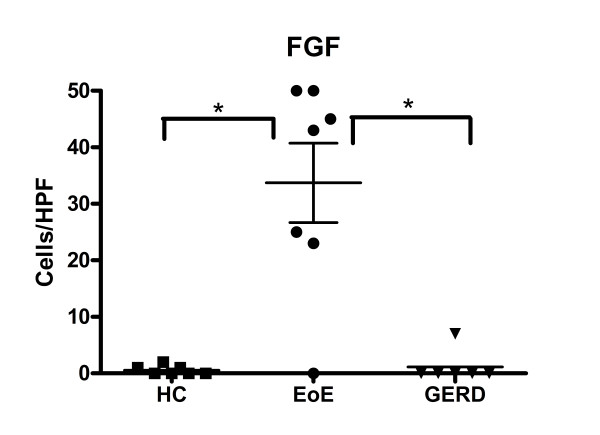
**The number of cells that are FGF positive cells are significantly greater in EoE subjects than in HC (p < 0.05) or GERD (p < 0.05)**. (* p < 0.05).

**Figure 5:**
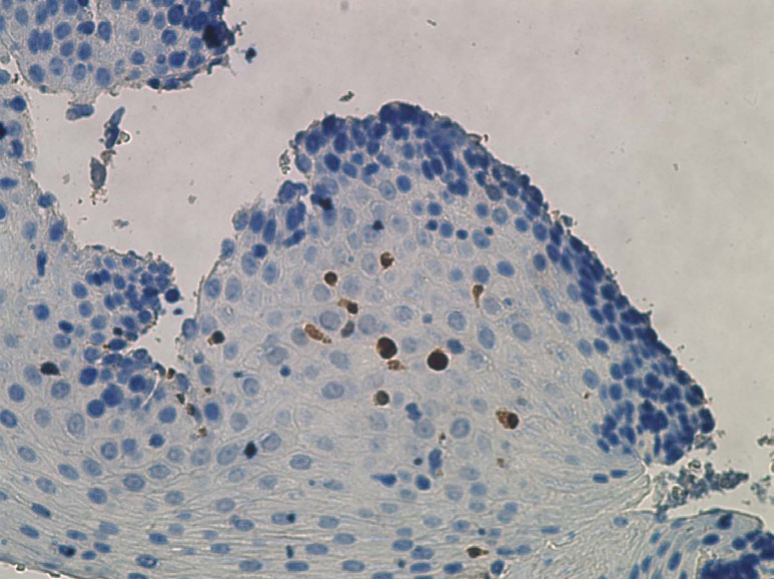
**IHC at 400× with FGF staining (representative EoE subject)**.

**Figure 6:**
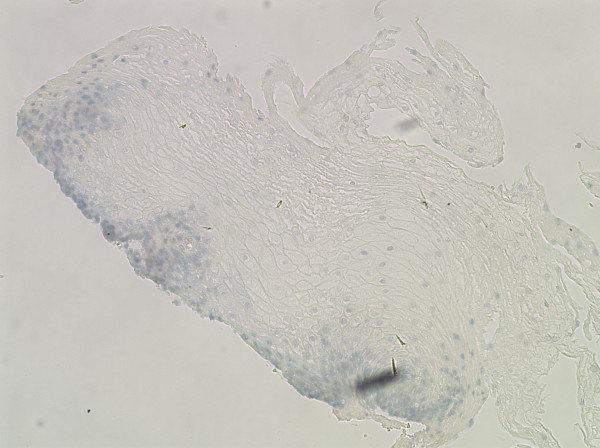
**IHC at 400× with FGF staining (representative GERD subject)**.

To test whether there were increases in expression of FGF in the local tissue of the esophagus in EoE compared to controls, immunohistochemistry (IHC) using the FGF antibody was conducted on slides from HC subjects (n = 7), GERD subjects (n = 6), and EoE subjects (n = 7). Cells were counted at 400× (Figure 4). EoE subjects were verified to have more than 15 eos/HPF as shown by EG2 antibody staining. HCs showed no cells surrounded by FGF, and there was only a minimal presence in GERD subjects. EoE subjects, however, consistently showed FGF in significantly higher counts compared to either GERD (p < 0.05) or HC (p < 0.05) (Figures 4, 5 and 6), based on an average of three areas per slide. The antibody was not guaranteed to be isoform-specific.

## Discussion

Our data indicate that fibroblast growth factor is among a set of factors that are differentially regulated in the periphery in EoE, suggesting that eosinophilic esophagitis is not only a local condition but also a systemic disorder that may be detected through analysis of plasma samples. Although symptoms of disease of may be localized at just the esophagus, our results show that EoE subjects have a set of unique peripheral immune indicators that could be used as diagnostic indicators. In addition, through analysis of PBMCs (which consist mainly of lymphocytes, monocytes, macrophages, and dendritic cells rather than granulocytes), we were able to detect an activation state present in other immune cells in EoE that could lead to subsequent activation of eosinophils. This would prove especially useful since current diagnosis involves an esophageal tissue biopsy, which carries potentially unnecessary risks.

The plasma fluid assay data provided here show that bFGF was upregulated in EoE subjects. Along with bFGF upregulation, an increase in eotaxin was also seen in EoE subjects. Interestingly, IL-5 was increased in the plasma of EoE subjects compared to that of GERD or HC. The subject with the highest IL-5 expression level did have the highest percent eosinophils in the blood (10.2%). Further work on the role of individual immune indicators vs. a composite biomarker tool using bFGF, eotaxin, and IL-5 simultaneously in blood samples from EoE vs. GERD vs. HC could be studied. Recent studies have also shown that IL-13 is overexpressed in the esophagus of EoE patients [20-22].

Recently, Mulder *et al*. found increased FGF-9 and FGF-Receptor expression levels in tissue from EoE subjects (n = 7) compared to HC (n = 7) and GERD (n = 7). However, there was no analysis of FGF expression in plasma or peripheral blood mononuclear cells. There was no evaluation of other FGF isoforms [23]. Moreover, a possible link to the mechanism of EoE pathology was still to be identified.

The fibroblast growth factor (FGF) family consists of 22 members that are essential to normal cell development, cell differentiation, and tissue repair after injury. The receptors for FGFs (FGFR) are tyrosine kinase receptors that are specific for particular FGF subsets and can be found in a variety of tissues, including epithelial and mesenchymal tissue [20]. bFGF increases the half-life of cells and could possibly increase the overall lifetime of the eosinophil in the esophagus [17]. Our data showing that the antiapoptotic pathways are associated with EoE confirm this possibility. Eosinophils are also known to secrete bFGF [23] and FGF-9 [24] upon the presence of necrotic epithelial cells. bFGF is also known to be secreted in case of cellular damage, such as the esophageal epithelial tissue damage experienced by EoE subjects [12]. Both eotaxin and bFGF mediate their biological effects through the ERK pathway [18,19]. Our data showed an increased expression of ERK in the PBMC of EoE subjects, and it is possible that, together with eotaxin, FGF enhances activation of eosinophils in EoE specifically.

The increased global expression of bFGF led to the investigation of the local expression bFGF of the esophageal biopsies of the subjects. Our data show that FGF, as assessed by IHC of local tissue and bFGF assessed by QT-PCR of PBMC, are upregulated in EoE. Initial immunohistochemistry antibody stains of FGF demonstrate an increase in local esophageal FGF expression levels in the lamina propria. FGF is diffusely spread over the lamina propria, close to the surface mucinous epithelium and covers mucosal glands as well. While healthy control and GERD subjects have very low expression of FGF, EoE subjects show higher FGF expression. It is important to note that bFGF and FGF-9 are highly homologous, sharing over 54% identical or similar amino acids (Protein BLAST). Since the FGF-9 antibody was made against the entire length of the amino acid, it is likely that homologous isoforms of FGF such as bFGF were also recognized. These data further suggested that FGF expression levels may help differentiate EoE from GERD, since EoE subjects have a significantly higher FGF expression level than GERD.

bFGF may be involved in the esophageal tissue fibrosis that is common to many EoE subjects. Past research has demonstrated that bFGF is a pro-fibrotic cytokine that may promote both fibrosis and angiogenesis by binding to the extracellular matrix [25,26]. The release of FGF by a wide variety of cells, especially in conditions of cellular damage, may explain the high levels of systemic FGF in EoE subjects. Additionally, FGF is irreversibly bound to the extracellular matrix after its release, further amplifying its fibrotic capabilities [26]. FGF may be further upregulated in the repair response after injury to the esophageal endothelium, leading to proliferation of fibroblasts and resulting fibrosis.

It has been shown that recurrent stricture formation and dysphagia can be associated with esophageal subepithelial fibrosis [27]. Studies on animal models have shown that stricture can form in the epithelium repair process, leading to an overly dense extracellular matrix, an overproduction of fibroblasts, and the development of scar tissue. In comparison to a previous study showing that EoE subjects have an increase in local FGF production [25], our study demonstrates that there is increase in FGF production locally and this increase is also reflected systemically. In addition, the extent of increase in FGF and ERK pathways was closely associated with the severity of the disease and endoscopic findings in the EoE patients; further patients will help determine if FGF and other immune indicators are correlated closely with EoE disease. Interestingly, we did not detect a difference in FGF and eotaxin expression in atopic (allergic) vs. non atopic EoE patients. However, further studies are needed to seek to identify possible pathological differences associated with atopic vs. non atopic EoE.

bFGF expression is essential to the transcription of ERK [18] and ERK feeds into the eotaxin-3 pathway, potentially further activating the eosinophil and improving its sensitivity and migration towards eotaxin in the esophageal tissue. Activation of ERK may induce both degranulation and chemotaxis of eosinophils [19]. It does appear that eosinophils also express bFGF [28]. The eosinophils may be operating in a positive feedback loop in which the expression bFGF is encouraging the increased activation of the eosinophil, which then leads to more bFGF expression and better chemotaxis. Additionally, like bFGF, FGF-9 can activate both ERK1 and ERK2 [29].

Further analysis of more subjects is necessary to identify additional immune indicators that constitute a unique EoE plasma protein composite or individual marker profile. Lower esophageal eosinophilia is common in GERD, and further determination of numbers of eosinophils in the tissues of GERD subjects is needed; we believe our current results reflect a relative difference in GERD and EoE subjects. We have focused on EoE to determine the involvement of specific blood immune indicators in the disease; we will continue to study other eosinophilic gastroenterological disorders such as eosinophilic gastroenteritis and eosinophilic colitis to determine the role of FGF in those disease entities.

We have demonstrated that fibroblast growth factors may play an important role in the pathophysiology of EoE and may be part of a set of immune indicators that could, without biopsy, differentiate EoE subjects from subjects with other clinically similar symptoms such as GERD. In this effort to determine possible pathological mechanisms, we found that FGF increase was associated with activation of ERK and of anti-apoptotic, pathways which could enhance eotaxin signaling and increase eosinophil lifespan, respectively.

## Conclusions

Subjects with eosinophilic esophagitis had different immune indicator profiles, specifically with increases in basic fibroblast growth factor in blood plasma, peripheral blood mononuclear cells, and local esophageal tissue compared to subjects with gastroesophageal reflux disease, ulcerative colitis, Crohn's disease, and healthy controls. The upregulation of fibroblast growth factor was found to be associated with ERK expression, which is in turn essential to the expression eotaxin-3, an eosinophil chemoattractant. Additionally, increases in basic fibroblast growth factor were found to be associated with activation of anti-apoptotic pathways. Therefore, FGF with eotaxin and antiapoptic factors could enhance migration and prolong the life-span of eosinophil, respectively. This may also explain the prolonged presence of higher than normal numbers of eosinophils in the esophagus.

## Competing interests

The authors declare that they have no competing interests.

## Authors' contributions

JH and JJ performed the plasma and PBMC separation, the immunohistochemistry, the QT-PCR tissue preparation, and the data analysis. JF, AP, TN, and SS aided in the immunohistochemistry. VS, CN, AQ, DB, WB, KC, JK, JP, and LN and KN aiding in obtaining biopsy and blood samples. NR and LH were involved in enrolling and consenting patients. JH drafted the manuscript. All authors read and approved the final manuscript.

## Supplementary Material

Additional file 1 **Supplementary tables**. Table S1a: Demographics of EoE patients. D (distal) and P (proximal) reflect location of the eosinophil count. Eosinophil counts are given as per high powered field (> greater than; = equal to). If negative for any allergies, total IgE less than 10 kU/mL. Table S1b: Demographics of GERD patients Table S1c: Demographics of healthy controls. Table S1 d. Demographics of Crohn's disease and ulcerative colitis patients. Table S2: Treatment emographics of EoE subjects. Click here for file
